# Mapping quantitative trait loci and developing their KASP markers for pre-harvest sprouting resistance of Henan wheat varieties in China

**DOI:** 10.3389/fpls.2023.1118777

**Published:** 2023-02-02

**Authors:** Cheng Kou, ChaoJun Peng, HaiBin Dong, Lin Hu, WeiGang Xu

**Affiliations:** ^1^ College of Agronomy, Northwest A&F University, Xianyang, China; ^2^ Institute of Crop Molecular Breeding, Henan Academy of Agricultural Sciences, Zhengzhou, Henan, China; ^3^ Henan Key Laboratory of Wheat Germplasm Resources Innovation and Improvement, Zhengzhou, Henan, China; ^4^ The Shennong laboratory, Zhengzhou, Henan, China

**Keywords:** wheat, pre-harvest sprouting, genome-wide association study, RNA-seq, KASP, mrMLM

## Abstract

**Introduction:**

Pre-harvest Sprouting (PHS) seriously affects wheat quality and yield. However, to date there have been limited reports. It is of great urgency to breed resistance varieties *via* quantitative trait nucleotides (QTNs) or genes for PHS resistance in white-grained wheat.

**Methods:**

629 Chinese wheat varieties, including 373 local wheat varieties from 70 years ago and 256 improved wheat varieties were phenotyped for spike sprouting (SS) in two environments and genotyped by wheat 660K microarray. These phenotypes were used to associate with 314,548 SNP markers for identifying QTNs for PHS resistance using several multi-locus genome-wide association study (GWAS) methods. Their candidate genes were verified by RNA-seq, and the validated candidate genes were further exploited in wheat breeding.

**Results:**

As a result, variation coefficients of 50% and 47% for PHS in 629 wheat varieties, respectively, in 2020-2021 and 2021-2022 indicated large phenotypic variation, in particular, 38 white grain varieties appeared at least medium resistance, such as Baipimai, Fengchan 3, and Jimai 20. In GWAS, 22 significant QTNs, with the sizes of 0.06% ~ 38.11%, for PHS resistance were stably identified by multiple multi-locus methods in two environments, e.g., AX-95124645 (chr3D:571.35Mb), with the sizes of 36.390% and 45.850% in 2020-2021 and 2021-2022, respectively, was detected by several multi-locus methods in two environments. As compared with previous studies, the AX-95124645 was used to develop Kompetitive Allele-Specific PCR marker QSS.TAF9-3D (chr3D:569.17Mb~573.55Mb) for the first time, especially, it is available in white-grain wheat varieties. Around this locus, nine genes were significantly differentially expressed, and two of them (TraesCS3D01G466100 and TraesCS3D01G468500) were found by GO annotation to be related to PHS resistance and determined as candidate genes.

**Discussion:**

The QTN and two new candidate genes related to PHS resistance were identified in this study. The QTN can be used to effectively identify the PHS resistance materials, especially, all the white-grained varieties with QSS.TAF9-3D-TT haplotype are resistant to spike sprouting. Thus, this study provides candidate genes, materials, and methodological basis for breeding wheat PHS resistance in the future.

## 1 Introduction

Wheat is a major worldwide food crop, and China is the largest wheat producer and consumer in the world. In 2022, Chinese wheat harvest area was 22,911.2 thousand hectares, and the total yield reached 135.76 million tons. In Henan, wheat harvest area and yield accounts for 24.8% and 28.1% in China, respectively, being the largest main wheat producing area in China. Its genetic improvement of wheat varieties has played an important role in its continuous improvement of wheat production capacity.

Pre-harvest Sprouting (PHS) refers to the phenomenon of seeds germinating and sprouting on the spike under rainy or humid conditions before wheat harvest. It is a worldwide natural disaster, and has been reported in China (Zhou et al., 2017), Japan (Kashiwakura et al., 2016), the United States (Nonogaki et al., 2014), Canada (Cabral et al., 2014), Europe (Rakoczy-Trojanowska et al., 2017), South Africa (Sydenham and Barnard, 2018), and Australia (Barrero et al., 2010). In China, the frequent and severe PHS spike hazards happened in the middle and lower reaches of Changjiang River winter wheat zone, southwest winter wheat zone, and northeast spring wheat zone (Jin, 1996; Zhang et al., 2010). In these zones, PHS resistance depends on dormant genes linked to red seed coat. In northern China, such as Henan, however, white-grained wheat varieties are used in production, and with the overall popularization of wheat mechanization harvest, that wheat should be harvested after being fully mature and dehydrated in the field. The varieties with PHS susceptibility have an increased probability of spike sprouting due to rainfall during the mature harvest period. Therefore, it is of great urgency to breed resistance varieties *via* PHS resistance quantitative trait nucleotides (QTNs) or genes of white-grained wheat.

Wheat PHS resistance is a complex quantitative trait controlled by multiple genes (Imtiaz et al., 2008). Thus, it is very important and necessary to identify these resistance loci and develop their molecular markers in crop breeding. In previous linkage analysis, a series of QTLs for PHS resistance has been located on all the 21 wheat chromosomes (Mohan et al., 2009; Cabral et al., 2014; Cao et al., 2016; Fakthongphan et al., 2016), in which repeatedly and stably QTLs were found on chromosome 3 (Kato et al., 2001; Osa et al., 2003; Kulwal et al., 2004; Mori et al., 2005; Liu and Bai, 2010). Currently, red-grained wheat varieties generally exhibit higher PHS resistance, because the PHS resistance genes on chromosomes 3A, 3B and 3D are thought to be closely linked to red seed coat, which is controlled by R dominant allele (Himi et al., 2011). Recently, genome-wide association studies (GWAS) have been used to identify QTLs and their candidate genes for wheat grain weight and plant height (Zanke et al., 2014; Chen et al., 2016; Wang et al., 2017), especially, Zhu et al. (2019) identified some QTLs and developed their molecular markers on wheat chromosomes 1AL, 3BS, and 6BL for PHS resistance, and Lin et al. (2017) identified two candidate genes for PHS resistance in 80 wheat varieties. However, the studies on wheat PHS resistance are relatively limited.

Chinese wheat local varieties showed higher PHS resistance than improved varieties (Wang et al., 2011; Liu et al., 2014), which provided valuable genetic resources for mining the loci of PHS resistance. In this study, 629 wheat varieties were measured for PHS resistance in 2020-2021 and 2021-2022, including 373 wheat local varieties over 70 years ago and 256 wheat improved varieties over the last 70 years in Henan Province, China. To mine some valuable QTNs for PHS resistance, these phenotypes were used to associate with SNP markers in the above 629 wheat varieties using several multi-locus GWAS methods. The results were validated by RNA-seq datasets between PHS resistance and susceptibility varieties, one confirmed QTN was used to develop Kompetitive Allele-Specific PCR (KASP) marker, and the KASP marker was further confirmed to be associated with PHS resistance. Thus, this study provides a valuable locus and white-grained wheat PHS resistance materials, which is available in main producing zones.

## 2 Materials and methods

### 2.1 Materials

In association mapping population, there were 629 Chinese wheat varieties, including 373 local wheat varieties from 70 years ago and 256 improved wheat varieties (lines). In autumns of 2020 and 2021, these varieties were planted in the experimental field of Henan Modern Agricultural Research and Development Base (East longitude: 113.707°, North latitude: 35.011°). The winter wheat varieties were provided by Institute of Crops Molecular Breeding, Henan Academy of Agricultural Sciences.

### 2.2 Measurement of PHS resistance in 629 wheat varieties

Based on the agricultural industry standards of the People’s Republic of China, NY/T 1939-2009, namely “standard” hereinafter, we harvested the varieties in the dough stage in turn, and 20 main stem spikes of each variety were stored in the refrigerator at -20°C. After all the varieties were harvested, we measured the PHS phenotypes of all the varieties on phytotron with temperature of 22°C ± 1°C and relative humidity of 95% ± 5%. Samples were removed from phytotron after 96 hours, and dried at 60°C for counting, spike sprouting (SS) was calculated from the formula x=(n/N)*100%,

where *n* is the number of sprouted grains per spike, and *N* is the total number of grains per spike. The relative SS index “*I*” of each variety to be tested was calculated from I=x1/x2.

where *x*_1_ is the SS of each variety to be tested, and *x*_2_ is the SS of the control variety, being Zhoumai 18 or a local variety with similar PHS phenotype with Zhoumai 18. Based on the criteria of PHS resistance in **Supplementary Table S1**, pre-harvest sprouting grade of each variety was determined.

### 2.3 Multi-locus GWAS for wheat PHS resistance in 629 varieties

As described in the reference (Du et al., 2021), all the 629 varieties were genotyped by wheat 660K microarray, and high quality genotypes of 314,548 SNP markers were obtained based on four screening criteria: alleles = 2, minor allele frequency (MAF) ≥ 0.01, missing ≤ 10%, and heterozygosity ≤ 10%. The best linear unbiased prediction (BLUP) values in 2020-2021 and 2021-2022 years was calculated by R language package (R 4.2.1). These marker genotypes were used to associate trait phenotypes or BLUP values in the 629 wheat varieties using the IIIVmrMLM (Li et al., 2022a; Li et al., 2022b) and mrMLM (Zhang et al., 2020) software packages, in which the latter included mrMLM (Wang et al., 2016), FASTmrMLM (Tamba and Zhang, 2018), FASTmrEMMA (Wen et al., 2017), pLARmEB (Zhang et al., 2017), ISIS EM-BLASSO (Tamba et al., 2017), and pKWmEB (Ren et al., 2018) methods. The population structure was determined using admixture_linux-1.3.0 software. The number of subgroups (K) was scanned from 2 to 5 using the admixture software and determined as two. The kinship matrix was calculated using the mrMLM software. The critical LOD score for significant QTLs was set as LOD = 3.0, which is equivalent to P-value = 2e-4. The Manhattan plots were drawn using the mrMLM software. The LD decay distance was calculated using vcftools v0.1.13, plink-v1.07, and PopLDdecay 3.41 softwares. The 2.192 Mb region was regarded as the upstream and downstream of a significant QTL.

### 2.4 Design and analysis of molecular markers for PHS resistance loci

0.2-0.3g fresh leaves were taken from each of 629 wheat varieties, pre-cooled with liquid nitrogen, crushed, and placed into 1.5mL centrifuge tube, and wheat genomic DNA was extracted based on Gawel and Jarret (1991).

#### 2.4.1 Design of KASP molecular marker for PHS resistance locus

The forward and reverse primers (FT: 5’-ATCAATTATCAGCTCTGGAT-3’; FC: 5’-ATCAATTATCAGCTCTGGAC-3’; R: 5’-AATCTTGACCTGTGTCCCGA - 3’) of KASP molecular markers were designed in the upstream 20 bp and downstream 155 bp according to the physical location information of significantly associated locus in reference to the Chinese spring sequence information of wheat Whole Genomics website (http://202.194.139.32/jbrowse-1.12.3-release/?data=Chinese_Spring1.0 ). HEX (red, 5’-GAAGGTCGGAGTCAACGGATT-3’) was added to the 5’ end of FT primer sequence and FAM (blue, 5’-GAAGGTGACCAAGTTCATGCT-3’) was added to the 5’ end of FC primer sequence, respectively. These primers were synthesized by Sangon Biotech (Shanghai) Co., Ltd. PCR reactions were performed in an Hydrocycler-thermal cycler in a total volume of 3μL, including 1.5μL KASP 2× Master Mix (LGC Technology (Shanghai) Co., Ltd.), 80 ng of template DNA, 0.06μL KASP Assay mix(100uM of Forward primer-FT, Forward primer-FC, Reverse primer-R and ddH2O mixed in a 12:12:30:46 volume ratio). PCR amplification were 94°C for 15min, 10 cycles of 94°C for 20s, 61°C-55°C for 60s by 0.6°C decrease per cycle, and with a final extension is 29 cycles of 94°C 20s, 55°C 60s.

#### 2.4.2 Sequence analysis of the KASP marker amplified product

The KASP molecular marker reaction products of Zhoumai 18 and Shengsimai were separated by 1% agarose gel electrophoresis, the target fragments were recovered and purified, which was cloned with pMDTM19-T vector (Takara Biomedical Technology (Beijing) Co., Ltd.), and sequenced by Sangon Biotech (Shanghai) Co., Ltd. At least 10 clones were sequenced for each variety. DNAMAN software was used to analyze the allelic variation of the amplified product sequences of KASP marker primers, and then BLAST (basic local alignment search tool) at EnsemblPlants database (http://plants.ensembl.org/index.html).

### 2.5 RNA-seq sample selection preparation and differential gene expression analysis

#### 2.5.1 RNA-seq sample selection preparation

According to the identification results of spike sprouting in association population, the highly resistant red-grained variety Shengsimai, the white-grained variety Baipimai, and the highly susceptible white-grained variety Zhoumai 18 were selected as RNA-seq samples. The sample processing method was carried out according to the standard. At the wax-ripening stage, all the three samples were cut from 15 cm below the spike, 9 spikes were taken from each material, which were divided into 3 portions, each spike was a biological replicate. And then, after soaking for 4 hours, one of 3 portions was taken out of liquid

Nitrogen and frozen for the 0-point control of RNA-seq (0h). The remaining two portions were further tested for PHS identification. A total of 96 hours were required for PHS identification. Samples were taken out and frozen in liquid nitrogen at 48 hours (48h) and 96 hours (96h). The subsequent RNA extraction library preparation, sequencing, and analysis results of RNA-seq were provided by Beijing Biomarker Technologies Co., Ltd.

#### 2.5.2 RNA-seq differential expression analysis of genes

Differential gene expression analysis of RNA-seq samples was performed on the website of Beijing Biomarker Technologies Co., Ltd. (http://www.biomarker.com.cn/). FDR < 0.05 was used as the standard for screening differentially expressed genes, and the difference groups were set according to the PHS resistance and susceptibility, and the PHS resistance of different seed coat colors, as shown in **Supplementary Table S2**. Using the differential gene expression datasets, the P-values were calculated by GO annotation enrichment tool of the Beijing Biomarker Technologies (https://international.biocloud.net/).

## 3 Results

### 3.1 Phenotypic analysis for PHS resistance in 629 wheat varieties

**Figure 1:**
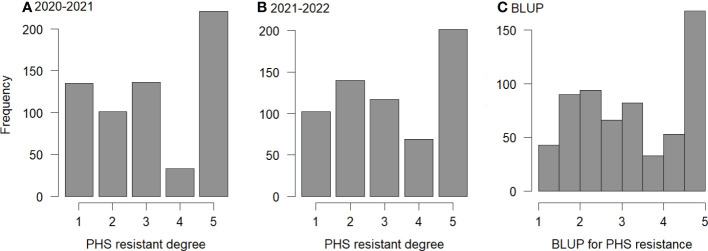
Frequency for PHS resistance. **(A–C)**: frequencies of PHS resistance for 629 varieties at 2020-2021, 2021-2022, and their BLUP values, respectively; 1 to 5: highly resistance, resistance, middle resistance, susceptibility, and highly susceptibility, respectively.

The spike sprouting method was used to identify the phenotypes of 629 wheat varieties (**Supplementary Table S3**), including 333 red-grained and 296 white-grained varieties. Among them, the numbers of red-grained varieties and white-grained varieties resistant to spike germination in the two years were 293 and 38, respectively. Red-grained varieties were generally more resistance than white-grained varieties. Among the 373 local varieties, 305 were red-grained varieties, 68 were white-grained varieties, and 298 were resistant to spike sprouting in both the two years. Among the 256 improved varieties, 28 were red-grained varieties, 228 were white-grained varieties, and 33 were resistant to spike sprouting in both the two years (**Figure 1**). This indicates that the grain color of wheat varieties in Henan Province has changed greatly from the local varieties before 1950 to the later improved varieties, and red-grained varieties were gradually changed to white -grained varieties. In the past two decades, all the varieties developed have been white-grained varieties.

In the identification of PHS resistance, the ranges of spike sprouting rates in 2020-2021 and 2021-2022 were 0.51%~99.22% (Mean ± SD: 35.38% ± 0.29%) and 0.00%~97.47% (Mean ± SD: 33.63% ± 0.25%), respectively, indicating abundant phenotypic variation in both environments. Analysis of variance showed that the spike sprouting rates were significant across genotypes, environments, and their interactions, and the heritability was 0.88 (*P*-value ≤ 0.001; **Supplementary Table S4**), indicating that wheat PHS resistance was mainly determined by genotypes and modified by environments.

### 3.2 GWAS for PHS resistance index in 629 wheat varieties

**Figure 2:**
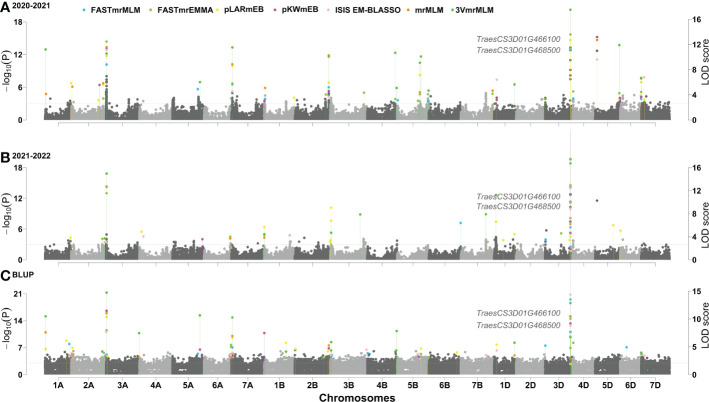
**(A–C)** Manhattan plot for PHS resistance index in 629 wheat varieties. If LOD score ≥ 10, the LOD scores obtained are transformed as LOD’ = 10 + (LOD – 10)/5.

The phenotypes for PHS resistance index in 629 wheat varieties in the two years were used to associate with all the SNP markers using six multi-locus GWAS approaches. As a result, a total of 22 QTNs were stably detected by multiple methods or environments, and their proportions of total phenotypic variation explained by each QTN (R^2^) was from 0.00001% to 38.1121% (**Table 1**). Among these loci, two loci, AX-95124645 on chromosome 3D and AX-109028892 on chromosome 5D, had been identified by Zhou et al. (2017), while other loci were identified for the first time, especially, AX-111020384 on chromosome 3A and AX-95124645 on chromosome 3D were identified by all the seven methods in the two software packages in all the two environments, and their R^2^ values were 12.8% and 38.1%, respectively (**Figure 2**), indicating the major QTN around AX-95124645 for wheat PHS resistance. As compared with the GWAS results for PHS resistance in 272 local varieties genotyped by Wheat660 SNP markers (Zhou et al., 2017), the resistance allele of AX-95124645 was found to be associated with only red-grained varieties in Zhou et al. (2017) and with both red-grained and white-grained varieties in this study. In linkage disequilibrium analysis, the LD decay distance in association mapping population was found to be 2.192Mb. This means that 2.192 Mb upstream and downstream regions of the significant QTL, that is QSS.TAF9-3D (chr3D:569.167Mb ~ 573.551Mb), may be used to mine candidate genes.

**Table 1:** Significant QTNs for PHS resistance index detected by multiple multi-locus GWAS methods in two environments.

QTN	Chr	Marker	Position (Mb)	Env	Methods	−log10(P-value)	R^2^ (%)	Reference
QTN1	1A	AX-110511933	12.859	3	2, 4	5.355~5.3664	0.5869~0.6868	
QTN2	1A	AX-109827872	545.852	3	3, 5	4.0032~6.3289	0.4481~1.0833	
QTN3	2A	AX-94559008	21.208	1, 3	1, 4	4.2459~6.2418	1.1445~2.3547	
QTN4	2A	AX-109841146	716.163	1	1, 2, 4	4.3164~6.8085	0.0652~1.6299	
QTN5	3A	AX-111020384	10.159	1, 2, 3	1~7	4.3263~36.0266	3.1246~12.8725	
QTN6	5A	AX-111670342	569.991	1, 3	3	4.4955~5.8588	0.00001~0.7568	
QTN7	6A	AX-110436229	590.075	2	1, 2	4.4267~4.7556	0.7937~1.1922	
QTN8	6A	AX-94617998	608.969	3	1, 2, 5	3.8013~5.7994	0.4255~0.7798	
QTN9	7A	AX-110492207	20.188	1, 3	1~5, 7	3.8284~24.2651	0.6686~3.1246	
QTN10	1B	AX-94741303	3.181	2, 3	1, 2, 4, 6	4.5796~8.3543	0.5791~1.886	
QTN11	2B	AX-111503288	765.578	1, 3	1, 4, 6	5.5209~12.389	1.4445~3.8593	
QTN12	3B	AX-111703196	16.805	2, 3	3, 4, 7	3.9937~9.9601	0.8401~1.7144	
QTN13	5B	AX-94487480	469.826	1, 3	2, 5	3.7531~4.4669	0.49~1.1933	
QTN14	5B	AX-108862465	511.697	1	1, 2, 4	5.009~8.1404	0.0551~1.318	
QTN15	5B	AX-108932221	536.054	3	2, 4	4.4563~5.4732	0.2389~0.3166	
QTN16	1D	AX-94392070	58.087	1, 3	4, 5, 6	4.0595~7.3964	0.915~1.557	
QTN17	1D	AX-110332164	458.942	2, 3	1, 4	4.1832~5.235	0.6609~0.905	
QTN18	3D	AX-95124645	571.359	1, 2, 3	1~7	5.0794~48.9107	4.9300~38.1121a	Zhou et al., 2017
QTN19	4D	AX-108916749	19.09	1, 3	2, 3	4.2911~6.551	0.00001~3.6386	
QTN20	5D	AX-109028892	45.711	1	1, 5, 6	10.6444~27.0823	7.2283~11.2516	Zhou et al., 2017
QTN21	6D	AX-109716798	143.583	3	3, 5	3.9008~5.7168	0.4118~0.5253	
QTN22	6D	AX-109293498	472.945	1, 3	1, 2, 3, 4, 6	4.2369~7.5779	0.5624~1.7955	

Env 1, 2, and 3: the PHS resistance indices in 2020-2021, 2021-2022, and their BLUP values, respectively. Methods 1 to 7: mrMLM, FASTmrMLM, FASTmrEMMA, pLARmEB, ISIS EM-BLASSO, pKWmEB, and IIIVmrMLM, respectively. “a”: the R^2^ value is greater than 30%.

### 3.3 KASP marker of QSS.TAF9-3D

**Figure 3:**
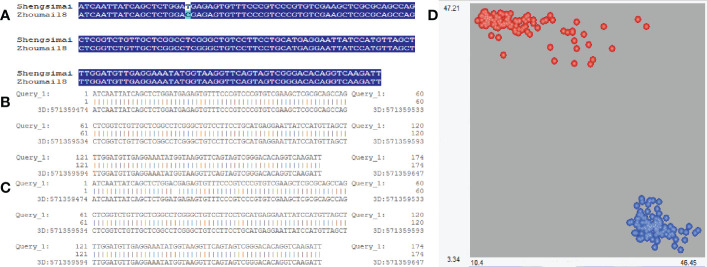
Sequence alignment of KASP marker amplification products at significant locus of QSS.TAF9-3D. **(A)** sequence alignment of the amplified products of Shengsimai and Zhoumai18; **(B)** Sequence alignment of Shengsimai amplification products from EnsemblPlants database; **(C)** Sequence alignment of Zhoumai18 amplification products from EnsemblPlants database; **(D)** Red and blue: varieties with QSS.TAF9-3D-TT and QSS.TAF9-3D-CC haplotypes, respectively. Horizontal and vertical coordinates represent the fluorescence signal values of FAM and HEX, respectively.

Using the KASP marker around a major QTN AX-95124645, two haplotypes were found in the 629 wheat varieties(**Figure 3**), namely QSS.TAF9-3D-TT and QSS.TAF9-3D-CC, which is completely consistent with the results of marker AX-95124645 obtained from 629 wheat varieties scanned by 660K chip. We also used T-A cloning and sequencing of the amplified products of Zhoumai18 (QSS.TAF9-3D-CC) and Shengsimai (QSS.TAF9-3D-TT), which there was only a T/C allele mutation at 26 bp in the amplified products of Zhoumai 18 and Shengmai. Using EnsemblPlants database (http://plants.ensembl.org/index.html), it was found that the above T/C alleles in the amplified products are exactly consistent with those at the physical location of marker AX-95124645 (**Figure 3**). QSS.TAF9-3D-TT and QSS.TAF9-3D-CC haplotypes could be distinguished by KASP molecular marker, having 261 QSS.TAF9-3D-TT haplotypes and 368 QSS.TAF9-3D-CC haplotypes in 629 wheat varieties.

The KASP marker was used to conduct haplotype analysis in 629 wheat varieties. The results were listed in **Supplementary Table S5**. The results showed that QSS.TAF9-3D-TT haplotype had significantly higher PHS resistance than QSS.TAF9-3D-CC haplotype. TAF9-3D-TT/CC markers accounted for 36.390% and 45.850% of phenotypic variation in SS_2021 and SS_2022, respectively. The QSS.TAF9-3D-TT haplotype was negatively correlated with the PHS resistance index, indicating that the QSS.TAF9-3D-TT haplotype was mainly distributed in varieties with high PHS resistance. Among 261 varieties with QSS.TAF9-3D-TT haplotype, 253 and 252 were resistant to spike sprouting in 2020-2021 and 2021-2022, respectively. Among the 38 white-grained resistant PHS varieties, 11 white grained varieties with QSS.TAF9-3D-TT showed PHS resistance (**Supplementary Table S6**). We considered that PHS resistance in the remaining 27 varieties was dependent on other related genes or QTLs.

### 3.4 RNA-seq analysis

#### 3.4.1 Validation of GWAS results by RNA-seq

Differentially expressed genes (DEGs) in QSS.TAF9-3D region were listed in **Table 2**. The results showed the existence of differential expressions between the PHS resistance varieties (Baipimai and Shengsimai) and the PHS susceptibility variety (Zhoumai18), indicating the association of QSS.TAF9-3D with PHS resistance. With the increase of treatment time, the number of DEGs between the two resistant varieties and one susceptible variety significantly increased. The number of DEGs in the two resistance varieties of Baipimai and Shengsimai with different seed coat colors increased first and then decreased with the increase of treatment time, indicating the association of seed coat color with PHS resistance.

**Table 2:** No. of differentially expressed genes in QSS.TAF9-3D region.

Time	DEG	Comparison		
point		Baipimai vs Zhoumai18	Shengsimai vs Zhoumai18	Baipimai vs Shengsimai
0h	Up	1	0	1
	Down	0	2	0
	Total	1	2	1
48h	Up	6	7	5
	Down	1	5	2
	Total	7	12	7
96h	Up	3	3	4
	Down	8	9	0
	Total	11	12	4

Time point indicates sample treatment time; DEG indicates differentially expressed genes, Up indicates that "vs" is less expressed in the former than in the latter, and Down indicates that "vs" is more expressed in the former.

### 4.1 Candidate genes around QSS.TAF9-3D

**Figure 4:**
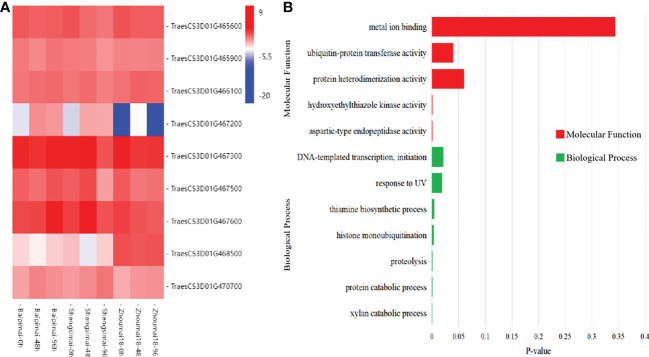
Differentially expressed genes in the QSS.TAF9-3D transcriptome. **(A)** heat map of differentially expressed genes (DEGs) between PHS resistant and susceptible. **(B)** GO Molecular Functions and Biological Process of DEGs.

In the region of QTL QSS.TAF9-3D, there were 56 genes. Using the RNA-seq datasets, 9 genes were found to be differentially expressed, as shown in **Figure 4**. Among them, TraesCS3D01G466100 GO annotation showed that it encodes ubiquitin protein transferase, and the NCBI conserved domain analysis showed that it encodes RING-type E3 ubiquitin ligase. In recent years, a large number of studies have shown that RING E3 is widely involved in abiotic stress processes (Cho et al., 2017). TraesCS3D01G468500 gene encodes initiation transcription factor TAF9. At present, the function of TAF9 has been reported in both human and yeast (Frontini et al, 2005; Knoll et al, 2020). However, TAF9 has limited studied in plants. Thus, the two genes were regarded as new candidate genes in this study.

## 4 Discussion

Genome-wide association studies for wheat PHS resistance in 629 local and improved varieties (lines) in Henan Province, China provide new insights into the genetic foundation of the important trait and variety breeding. In previous studies, most of them focused on the PHS resistance in southwest and southern wheat zones in China, for example, Zhou et al. (2017) found that the landraces in Chinese wheat zones with high precipitation showed strong PHS resistance in 717 Chinese wheat landraces, but there were few studies on PHS resistance in northern wheat zones with less rain. In this study, 373 local varieties before 1950 and 256 improved varieties after 1950 in Henan Province were included. It was found that genes for wheat PHS resistance were gradually lost in the process of selection and breeding for yield, quality, and other important breeding traits. The main reason is that most of the loci or genes for wheat PHS resistance are found to be linked with red seed coat, while most improved varieties are white grained, resulting in the generally reduced PHS resistance of modern improved varieties. In this study, 38 white grain resistant varieties were observed, and this study provides a material basis for breeders to select white-grained resistant varieties for PHS.

Zhou et al. (2017) found three major PHS resistant QTLs on chromosomes 3A, 3D, and 5D, in which the marker AX-95124645 was located on Chr 3D. In this study, AX-95124645 locus was identified by several multi-locus methods in two environments to be associated with PHS resistance, especially, its R^2^ value was 38%. However, this study provides three new results compared with previous studies. First, resistant and susceptible PHS varieties were used to conduct RNA-seq analysis, and 9 DEGs were found in the 2.192 Mb upstream and downstream intervals of AX-95124645, and two candidate genes were predicted. Then, the KASP marker QSS.TAF9-3D-TT/CC was developed based on the AX-95124645 locus. The results showed that QSS.TAF9-3D-TT/CC haplotypes with only one T/C base allele variation could completely distinguish all the PHS resistant and susceptible varieties. Finally, all the QSS.TAF9-3D-TT haplotypes were found in 11 white-grained varieties to be resistant for PHS.

It should be point out that the KASP marker QSS.TAF9-3D developed in this study is valuable. First, the KASP marker was used to select 11 white-grained resistant varieties with excellent haplotype QSS.TAF9-3D-TT, indicating its possibility of marker-assisted selection in white-grained varieties for PHS resistance. But among 629 varieties, the numbers of white-grained varieties resistant to spike germination in the two years was 38. Because Wheat PHS resistance controlled by multiple genes (Imtiaz et al., 2008), so we consider the spike sprouting resistance of the remaining 27 white grain varieties was caused by other genes or QTLs. Second, this marker uses high-throughput KASP genotyping technology. In particular, KASP is based on conventional PCR and fluorescence detection, which can meet the requirements of low, medium, and high throughput genotyping on the basis of ordinary laboratory operation (Semagn et al., 2014), indicating that it is flexible, cheap, high-throughput, automated, and accurate. As we known, KASP, as an alternative to TaqMan, is similar in principle to TaqMan (also based on terminal fluorescence reading), but it differs from TaqMan technology in the following ways. It uses a universal probe, which can be used with a variety of different gene-specific primers, without the need for probe synthesis for each specific site, which greatly reduces the reagent cost of the experiment (Majeed et al., 2018). In conclusion, QSS.TAF9-3D-TT/CC markers can be used for higher throughput and more accurate screening of PHS resistance varieties, especially in white-grained varieties, which provides a strong theoretical basis for molecular mark-assisted breeding.

Myb10 is an important regulatory gene in the pathway of pigment synthesis. The earliest MYB-type transcription factor identified was maize Colorless 1 (Paz-Ares et al., 1987). In wheat, *Tamyb10* gene is believed to be related to seed dormancy, because it may affect the sensitivity of wheat embryo to ABA. Lang et al. (2021) found that *myb10-D* gene, as a candidate gene for PHS-3D, not only regulates the synthesis of flavonoid compounds, but also increases the ABA concentration in developing seeds, thus inhibiting the wheat PHS. In this study, the candidate gene TraesCS3D01G468400 was found to be consistent with *Tamyb10-D* in the annotation information of 61 genes in the QSS.TAF9-3D region. Although Himi et al. (2011) designed the *Tamyb10-D* marker to screen PHS resistance materials, *Tamyb10-D* is an important regulatory gene involved in the pigment synthesis of wheat seed coat so that its corresponding molecular marker is mainly used to screen the PHS resistance of red-grained wheat varieties, indicating its difficulty in the application of white-grained varieties. We identified two differentially expressed genes TraesCS3D01G466100 and TraesCS3D01G468500 in the QSS.TAF9-3D region using RNA-seq. TraesCS3D01G466100 GO annotation shows that it encodes C3HC4-RING fifinger E3 ubiquitin ligase. Yang et al. (2016) identified *AtAIRP4* in Arabidopsis, which is induced by ABA and other stress treatments. *AtAIRP4* encodes a cellular protein with a C3HC4-RING finger domain in its C-terminal side, which has *in vitro* E3 ligase activity. A large number of studies have shown that the dormancy period of wheat seeds is negatively correlated with the degree of PHS (Flintham et al., 2000; Biddulph et al., 2008; Shu et al., 2016), and ABA plays a crucial role in promoting seed dormancy and inhibiting seed germination (Martínez-Andújar et al., 2011). Thus, it is possible for *TraesCS3D01G466100* to affect PHS resistance by regulating seed ABA levels. *TraesCS3D01G468500* gene encodes the initiation transcription factor TAF9. Yang (2015) cloned a gene *CpTAF9* in the woody ornamental plant *Chimonanthus melanoides*. Salt stress, high temperature or ABA application promoted the expression of *CpTAF9* gene in leaves. ABA is an important hormone regulating seed dormanness. *TraesCS3D01G468500* gene may affect wheat spike germination by indirectly regulating seed ABA content. We selected these two genes as new PHS resistance candidate genes.

## 5 Conclusion

We firstly identified 38 white-grained varieties with PHS resistance in 629 wheat varieties (lines) from Henan Province, China, stably identified a major QTN AX-95124645 on chromosome 3D, and developed its KASP marker QSS.TAF9-3D-TT/CC. This marker haplotype can effectively detect the PHS resistance materials, especially, all the white-grained varieties with QSS.TAF9-3D-TT haplotype are resistant to spike sprouting, which can be used for molecular mark-assisted breeding of spike sprouting resistance in white-grained varieties. This study provides material and methodological basis for breeding wheat PHS resistance in the future.

## Data availability statement

The sequencing data have been successfully submitted to the GEO database and applied for public disclosure. The GEO accession number is GSE222342, and the BioProject accession number is PRJNA919175.

## Author contributions

W-GX conceived and managed the research and revised the manuscript. CK and C-JP analyzed datasets. CK measured the phenotypes of the traits. LH and H-BD provided the research materials and Instruments. CK wrote the draft. All authors contributed to the article and approved the submitted version.
