# Supplementary material for: Mapping quantitative trait loci and developing their KASP markers for pre-harvest sprouting resistance of Henan wheat varieties in China
Source: Front Plant Sci. 2023 Feb 2;14:1118777. doi: 10.3389/fpls.2023.1118777 (PMC9976778; doi:10.3389/fpls.2023.1118777)
Supplement: Supplementary file 1 [file DataSheet_1.pdf]

Table S1. The criteria for wheat PHS resistance

PHS resistance	Relative SS index "I"	Degree
Highly Resistance (HR)	< 0.05	1
Resistance (R)	0.05~0.20	2
Middle Resistance (MR)	0.21~0.40	3
Susceptibility (S)	0.41~0.60	4
Highly Susceptibility (HS)	> 0.60	5

Table S2. Differential grouping settings for RNA-seq

Time (hour)	Differential grouping		
	**Shengsimai vs Zhoumai18**	**Baipimai vs Zhoumai18**	**Baipimai vs Shengsimai**
0	Shengsimai-0h vs Zhoumai18-0h	Baipimai-0h vs Zhoumai18-0h	Baipimai-0h vs Shengsimai-0h
48	Shengsimai-48h vs Zhoumai18-48h	Baipimai-48h vs Zhoumai18-48h	Baipimai-48h vs Shengsimai-48h
96	Shengsimai-96h vs Zhoumai18-96h	Baipimai-96h vs Zhoumai18-96h	Baipimai-96h vs Shengsimai-96h

**: The 0.001 probability level of significance.

Table S3. PHS resistance grades of 629 wheat varieties

Number	Name of variety	QSS.TAF9-3D	SS_2021	SS_2022	Seed color
HXZ1	Fumai	CC	3	4	white
HXZ2	Qitoufumai(Shanxian1)	CC	2	4	white
HXZ3	Qisiniu	TT	3	3	white
HXZ4	Baixiantiao	CC	2	2	white
HXZ5	Baifumai	CC	2	3	white
HXZ6	Erjimai	CC	5	5	red
HXZ7	Baiheshangtou(Jiaxian)	CC	2	5	white
HXZ8	Huangguaxian(Yuxian)	CC	2	3	white
HXZ9	Yaerzui	TT	3	3	white
HXZ10	Heshangtou(Anyang)	TT	3	3	white
HXZ11	Baiyupi	TT	5	5	red
HXZ12	Shuanghoumai	TT	3	3	white
HXZ13	Zhuganqing(Tangxian)	TT	3	3	white
HXZ14	Baihulutou	TT	3	3	white
HXZ15	Xiaobaimai(Mianchi)	TT	3	3	white
HXZ16	Huitucao	TT	2	3	red
HXM17	Ziganyangmai	CC	1	2	red
HXM18	Baitiaoyu(Yiyang)	CC	1	2	red
HXM19	Dazihong(Yancheng)	CC	1	3	red
HXM20	Baiheshangtou(Nanyang)	CC	2	4	red
HXM21	Zhuaziya	CC	2	2	red
HXM22	Dajiantou	CC	1	2	red
HXM23	Tiepachimai(Wuzhi1)	CC	1	1	red
HXM24	Xiaofoushou(Jiyuan1)	TT	NA	1	red
HXM25	Sanyuehuang(Wenxian1)	TT	1	2	red
HXM26	Baitutou	TT	2	2	red
HXM27	Tuhuloutou	TT	2	3	red
HXM28	Dakoumai	CC	5	5	white
HXM29	Guangtoubaisanmai	CC	5	5	white
HXM30	Huixianhong	CC	5	5	red

HXM31	Sanyuehuang(Wenxian2)	CC	4	5	white
HXM32	Sanyuehuang(Mengxian)	CC	4	5	red
HXM33	Huoshaomai	CC	4	4	white
HXM34	Zhuganqing	CC	5	5	red
HXM35	Jinguoyin(Lingbao)	CC	5	5	red
HXM36	Mazhamai	CC	3	5	red
HXM37	Hongheshangtou(Baofeng)	TT	2	2	red
HXM38	Hongheshangtou(Nanyang1)	TT	3	5	red
HXM39	Honggoudou	CC	2	2	red
HXM40	Yangmai	CC	1	2	red
HXM41	Baiyintiao	CC	3	4	white
HXM42	Zigancao(Yichuan)	CC	3	3	white
HXM43	Baimai(Xichuan)	CC	3	3	white
HXM44	Baimai(Xiangcheng)	CC	2	3	white
HXM45	Baipimai	TT	1	3	white
HXM46	Xiaomanshu	CC	3	4	red
HXM47	Zaosantian	CC	2	5	red
HXM48	Sanyuehuang(Nanle)	CC	3	4	red
HXM49	Ziganbaimangxian	CC	3	2	red
HXM50	Liulinger	CC	5	5	red
HXM51	Xiaobaimang(Puyang)	CC	3	5	red
HXM52	Dabaimai	CC	2	3	red
HXM53	Wuhuatou(Fengqiu)	CC	2	3	red
HXM54	Baimangcao(Tongxu)	CC	3	2	red
HXM55	Duangancao(Zhongmu)	CC	1	1	red
HXM56	Baimangcao(Kaifeng)	CC	3	4	white
HXM57	Baitiaoyu(Mixian)	CC	3	3	white
HXM58	Gedacao(Luanchuan)	TT	2	2	red
HXM59	Youzitou	TT	1	2	red
HXM60	Laolaiqiao	TT	1	2	red
HXM61	Xiaozibai(Lushan)	TT	1	2	red
HXM62	Hongboyan(Yuxian)	TT	3	2	red

HXM63	Yulincao(Zhenping)	TT	3	1	red
HXM64	Gedacao(Fangcheng)	TT	2	2	red
HXZ65	Yanghuomai	TT	3	1	red
HXZ66	Nvmai	TT	2	1	red
HXZ67	Bodijiang(Xichuan)	TT	2	2	red
HXZ68	Baimangcao(Dengxian)	CC	3	2	red
HXZ69	Kumai(Dengxian)	TT	2	2	red
HXZ70	Baihuomai	TT	3	1	red
HXZ71	Meiguocao	TT	2	1	red
HXZ72	Dachuantiaomai	TT	2	2	red
HXZ73	Shengsimai	TT	1	1	red
HXZ74	Jiyulin	TT	1	1	red
HXZ75	Duanganchaoxiaomai	TT	1	1	red
HXZ76	Sinenggan	TT	1	4	red
HXZ77	Wugongcao(Xinxian)	TT	2	1	red
HXZ78	Jinbangchui	TT	1	2	red
HXZ79	Duangancao(Luoshan)	TT	1	2	red
HXZ80	Datoucao(Guangshan)	TT	1	2	red
HXM81	Lvmangcao	TT	1	2	red
HXM82	Manmai	TT	1	2	red
HXM83	Silengmai	TT	1	3	red
HXM84	Bagucha	TT	2	3	red
HXM85	Shuilaomai	TT	3	2	red
HXM86	Datoucao(Gushi)	TT	1	1	red
HXM87	Changshengmai	TT	3	2	red
HXM88	Wugongcao(Xincai)	TT	3	3	red
HXM89	Xiaozihong	TT	3	2	red
HXM90	Datouben(Runan)	TT	3	2	red
HXM91	Jiuyisi	TT	3	3	red
HXM92	Changsuimai	TT	3	3	red
HXM93	Datouben(Qinyang1)	TT	1	3	red
HXM94	Datouben(Queshan)	TT	2	2	red

HXM95	Yaozhouhong	TT	3	2	red
HXM96	Baibenmang	CC	1	2	red
HXM97	Ziganbai	CC	3	4	white
HXM98	Laohongpi	TT	2	3	red
HXM99	Jinzihong	TT	3	2	red
HXM100	Baidadu	TT	1	2	red
HXM101	Wuhuatou(Zhongmu)	TT	2	3	red
HXM102	Helubai	CC	4	5	white
HXM103	Hongmangbai(Xixian)	CC	3	4	red
HXM104	Daizimai	CC	3	4	white
HXM105	Xiaohongmang(Puyang)	CC	5	5	white
HXM106	Fanglutou	CC	5	3	white
HXM107	Shizitou	CC	5	4	white
HXM108	Huangguaxian(Kaifeng)	CC	4	5	white
HXM109	Dazipang	CC	5	4	white
HXM110	Hongmanghong(Zhongmu)	CC	2	5	red
HXM111	Huaiqingfumai	TT	2	2	red
HXM112	Hongdanvmai	TT	3	3	red
HXM113	Jinsihuang	TT	1	3	red
HXM114	Laoliangkou	TT	3	3	red
HXM115	Hongcantiao	TT	1	1	red
HXM116	Hongmanghongmai(Yanling)	TT	1	2	red
HXM117	Zaobanyue(Zhenping)	TT	2	2	red
HXM118	Huolibeng	TT	3	1	red
HXM119	Yixiaomai	TT	2	2	red
HXM120	Qiuzimai(Shangcheng)	TT	1	3	red
HXM121	Erxiaomai	TT	3	2	red
HXM122	Zaobanyue(Dengxian)	TT	1	3	red
HXM123	Huomai(Xichuan)	TT	2	4	red
HXM124	Hongyouzi(Xichuan)	TT	2	2	red
HXM125	Hongjuanmang	TT	3	2	red
HXM126	Xianmai	TT	1	2	red

HXM127	Huomai(Xinyang)	TT	4	2	red
HXM128	Sanyuehuang(Gushi)	TT	1	2	red
HXM129	Kuloucao	TT	1	1	red
HXM130	Hongsuihong	TT	1	3	red
HXM131	Sanhongmai	TT	1	3	red
HXM132	Dazihong(Shangcai)	TT	2	2	red
HXM133	Hongmanghongmai(Nanle)	TT	1	1	red
HXM134	Jiandihong	TT	2	3	red
HXM135	Hongjianmai(Fengqiu)	TT	1	1	red
HXM136	Jianmai(Fengqiu)	TT	1	3	red
HXM137	Hongjianmai(Yucheng)	TT	2	1	red
HXM138	Jianmai(Shangqiu)	TT	2	3	red
HXM139	Qiuzimai(Shangqiu)	TT	1	2	red
HXM140	Kaoshanhong(Shangqiu)	TT	1	2	red
HXM141	Wuhuatou(Suixian)	TT	3	3	red
HXM142	Dazibai(Suixian)	TT	1	2	red
HXM143	Kaoshanhong(Zhecheng)	CC	3	2	red
HXM144	Jianmai(Kaifeng)	TT	NA	3	red
HXM145	Hongboyan(Zhongmu)	TT	3	2	red
HXM146	Baihulu	CC	3	5	red
HXM147	Silengmai(Zhecheng1)	CC	2	1	red
HXM148	Laochushanbao	TT	3	5	red
HXM149	Huisancao	CC	1	3	red
HXM150	Siyuecao	TT	1	2	red
HXM151	Duangancao(Xinyang)	TT	2	2	red
HXM152	Banjiemang(Runan)	CC	1	2	red
HXM153	Duanganzhuganqing	CC	1	2	red
HXM154	Hongheshangtou(Nanyang2)	TT	2	2	red
HXM155	Liekoucao	TT	1	1	red
HXM156	Shajiangbai	TT	1	1	red
HXM157	Yangguangtou	CC	1	3	red
HXM158	Jiacangmai	TT	1	1	red

HXM159	Quanmangmai(Yuxian)	TT	1	3	red
HXM160	Xiaoquanmang(Xiangcheng)	TT	3	2	red
HXZ161	Daquanmang	TT	1	2	red
HXZ162	Liangguangtou	TT	3	3	red
HXZ163	Tiepachimai(Wuzhi2)	TT	3	2	red
HXZ164	Baijiantou	TT	1	1	red
HXZ165	Shandatou	TT	3	2	red
HXZ166	Heshangtou(Zhenping)	TT	3	2	red
HXZ167	Siyixiaomai	TT	2	3	red
HXZ168	Xinpeibai	TT	1	2	red
HXZ169	Baimai(Lushan1)	CC	3	5	white
HXZ170	Ermangmai(Qinyang)	CC	2	3	white
HXZ171	Kumai(Mengxian)	TT	1	1	red
HXZ172	Chushanbao(Xinan)	TT	1	1	red
HXZ173	Yinghuinong	TT	1	1	red
HXZ174	Baimayidan	TT	3	2	red
HXZ175	Lengmai	TT	1	2	red
HXZ176	Yuercao	CC	3	2	red
HXM177	Quanmangmai	TT	2	2	red
HXM178	Daquanmai	TT	1	2	red
HXM179	Cantiaomai(Zhenping)	TT	1	1	red
HXM180	Baijuanmang	TT	3	1	white
HXM181	Deguohong	TT	1	2	red
HXM182	Kuquanmang	TT	1	1	red
HXM183	Hongyouzitou(Linru)	TT	3	2	red
HXM184	Hongqulianmang	CC	1	2	red
HXM185	Chushanbao(Songxian1)	TT	1	1	red
HXM186	Qitoufumai(Shanxian2)	TT	1	2	red
HXM187	Cantiaomai(Luanchuan)	TT	1	2	red
HXM188	Huisanbao	TT	1	2	red
HXM189	Bodijiang(Lushi)	TT	1	2	red
HXM190	Juanmaohu	TT	1	2	red

HXM191	Duanermang	TT	1	3	red
HXM192	Pishanba	TT	1	3	red
HXM193	Ermangmai(Nanzhao)	TT	3	2	red
HXM194	Ermangmai(Zhenping)	TT	1	3	red
HXM195	Ermangmai(Fangcheng)	TT	1	2	red
HXM196	Lvweibamai	TT	1	3	red
HXM197	Hongquanmang	CC	5	3	white
HXM198	Zhangfeihu	CC	5	5	white
HXM199	Wangshimai	TT	2	2	red
HXM200	Saogudan	CC	1	2	red
HXM201	Youmai	CC	2	1	red
HXM202	Baisuimai	CC	5	5	red
HXM203	Lengdingzi	CC	1	3	red
HXM204	Silengmai(Zhecheng2)	CC	5	5	white
HXM205	Baimangmai(Xiangcheng1)	TT	1	2	red
HXM206	Dawangshuibai	TT	2	3	red
HXM207	Kumai(Zhenping)	TT	1	1	red
HXM208	Youzitou(Suiping)	TT	3	3	red
HXM209	Youzitou(Runan)	TT	2	1	red
HXM210	Xiadanba	CC	3	4	white
HXM211	Shiganmai	CC	3	3	white
HXM212	Bolanmai	CC	1	3	red
HXM213	Xiadanba	CC	1	3	red
HXM214	Fenzhixiaomai	CC	1	3	white
HXM215	Foshoumai	CC	2	4	white
HXM216	Dongmai	CC	3	3	white
HXM217	Jiutoumai	CC	3	4	white
HXM218	Wuzitoumai	CC	3	3	white
HXM219	Jinsita	CC	3	3	white
HXM220	Quermang	TT	2	4	red
HXM221	Youmangsaogudan	CC	5	5	red
HXM222	Hongmai	CC	1	3	red

HXM223	Gedacao(Xiangcheng1)	TT	1	2	red
HXM224	Baishapi	TT	1	2	red
HXM225	Hongmangbai(Zhecheng)	CC	4	5	white
HXM226	Bodanbamai	CC	5	5	white
HXM227	Honghulutou	CC	3	3	white
HXM228	Hongyouzitou(Nanle)	CC	2	1	red
HXM229	Baimangyouzi	CC	1	3	red
HXM230	Youzimai(Qingfeng)	CC	4	4	white
HXM231	Xiaobaimang(Xunxian)	TT	1	1	red
HXM232	Hanbadan	CC	4	5	white
HXM233	Yududasuibai	CC	4	3	white
HXM234	Kumai(Nanyang)	TT	1	2	red
HXM235	Laolaihong	TT	2	1	red
HXM236	Xiaofoshou(Jiyuan2)	CC	2	3	red
HXM237	Hongtutou(Jiyuan)	CC	4	5	white
HXM238	Baigedatou	CC	2	3	white
HXM239	Fangtoumai(Xiuwu)	CC	3	3	red
HXM240	Banjiemang(Xiuwu)	TT	3	3	red
HXM241	Youzimai(Fengqiu)	CC	5	3	white
HXM242	Xiaohongmang(Zhengzhou)	CC	3	2	red
HXM243	Hongqumang	CC	1	1	red
HXM244	Zaobanyue(Xichuan)	TT	1	2	red
HXM245	Hanzaobadou	CC	4	3	red
HXM246	Hongtutou(Lankao)	TT	4	3	red
HXM247	Toulongbei	CC	2	1	red
HXM248	Tutoumai(Qixian)	CC	2	3	white
HXM249	Hongmanghong(Zhecheng)	TT	1	2	red
HXM250	Misuimai	CC	1	2	red
HXM251	Sanyuehuang(Luoyang)	TT	1	1	red
HXM252	Laohongmai	TT	1	1	red
HXM253	Chushanbao(Songxian2)	TT	2	2	red
HXM254	Yingsizhuang(Songxian)	TT	1	3	red

HXM255	Sanyuehuang(Lushi)	CC	5	3	white
HXM256	Zhiliaomai	TT	2	3	red
HXZ257	Xiaosanyuehuang	TT	3	2	red
HXZ258	Baigeda(Lushi)	CC	5	4	white
HXZ259	Bodijiang	TT	5	2	red
HXZ260	Huihuicao	TT	3	4	red
HXZ261	Huomai(Lushi)	TT	1	2	red
HXZ262	Bensiyuehuang	TT	1	1	red
HXZ263	Heshangtou(Luanchuan)	TT	2	1	red
HXZ264	Honggancao	TT	3	1	red
HXZ265	Xiaobaimai(Lingbao)	CC	3	1	red
HXZ266	Baigeda(Lingbao)	CC	2	2	red
HXZ267	Hongheshangtou(Lingbao)	TT	2	2	red
HXZ268	Jinguoyin(Sanmenxia)	CC	3	1	red
HXZ269	Yingsizhuang(Yichuan)	TT	3	1	red
HXZ270	Yingbazhuang	TT	2	1	red
HXZ271	Quanyangmai	TT	1	2	red
HXZ272	Huangguaxian(Yancheng)	CC	5	4	white
HXM273	Xiaozibai(Yuxian)	CC	5	3	white
HXM274	Hongmangbaimai	CC	5	5	white
HXM275	Hongheshangtou(Qinyang)	TT	2	3	red
HXM276	Ermangmai(Qinyang2)	CC	2	1	red
HXM277	Mazhamei	TT	1	2	red
HXM278	Datouben(Qinyang2)	TT	3	2	red
HXM279	Sanbaimai	CC	3	4	white
HXM280	Yumai(Pingyu)	CC	1	1	red
HXM281	Youzimai(Pingyu)	TT	1	2	red
HXM282	Hongmangbai(Shangcai)	CC	5	3	white
HXM283	Youzimai(Shangcai)	TT	1	2	red
HXM284	Wangshuibai	TT	3	3	red
HXM285	Wangshanbai	TT	3	3	red
HXM286	Baiheshangtou(Xinxian)	TT	1	2	red

HXM287	Ercaomai(Huaiyang)	TT	1	1	red
HXM288	Simangmai	TT	1	1	red
HXM289	Youzimai(Taikang)	TT	3	1	red
HXM290	Hongsuibai	CC	5	5	white
HXM291	Zigancao(Shenqiu)	TT	3	1	red
HXM292	Hongmangmai(Nanyang)	TT	2	1	red
HXM293	Zhuganqing(Nanyang)	TT	3	2	red
HXM294	Datouhong(Neixiang)	TT	2	2	red
HXM295	Hongyouzi(Zhenping)	TT	2	2	red
HXM296	Hongmangmai(Fangcheng)	TT	3	2	red
HXM297	Pushanbamai	TT	1	2	red
HXM298	Hongquanmang(Dengxian)	CC	1	2	red
HXM299	Datouhong(Dengxian)	TT	3	2	red
HXM300	Yumai(Dengxian)	CC	5	4	white
HXM301	Hongquanmang(Xixia)	TT	3	2	red
HXM302	Yumai(Tanghe)	CC	1	1	red
HXM303	Ermangmai(Lushan)	TT	3	1	red
HXM304	Ermangmai(Yexian)	TT	3	3	red
HXM305	Ercaomai(Xuchang1)	TT	1	3	red
HXM306	Ercaomai(Xuchang2)	TT	1	1	red
HXM307	Sanyuehuang(Linying)	TT	2	1	red
HXM308	Sanyuehuang(Yexian)	TT	1	1	red
HXM309	Sanmangmai	TT	3	1	red
HXM310	Sanbaoxian	CC	3	3	white
HXM311	Damai	TT	3	1	red
HXM312	Dazibai(Changge)	CC	NA	4	white
HXM313	Dazihong(Xuchang)	TT	1	1	red
HXM314	Dazicao(Xuchang)	TT	3	1	red
HXM315	Dazicao(Yexian)	TT	1	1	red
HXM316	Xiaoguangtou	TT	1	2	red
HXM317	Xiaozibai(Xucheng)	CC	3	3	red
HXM318	Xiaozicao	TT	3	1	red

HXM319	Xiaoquanmang(Linying)	TT	3	1	red
HXM320	Wuhuatou(Linying)	TT	1	1	red
HXM321	Wuhuatou(Xihua1)	TT	2	2	red
HXM322	Wuhuatou(Xihua2)	TT	3	2	red
HXM323	Changzicao	CC	1	1	red
HXM324	Wukelan	TT	3	1	red
HXM325	Yubianmai	TT	2	1	red
HXM326	Baimanghong(Yanling)	TT	3	1	red
HXM327	Baimanghong(Xihua1)	TT	2	1	red
HXM328	Baimanghong(Xihua2)	TT	3	1	red
HXM329	Baimanghong(Xihua3)	TT	2	1	red
HXM330	Baimanghong(Yexian)	TT	2	1	red
HXM331	Baimangmai(Xiangcheng2)	TT	1	3	red
HXM332	Baimai(Linying)	CC	2	4	red
HXM333	Baimai(Yexian)	TT	1	1	red
HXM334	Baimai(Lushan2)	TT	2	2	red
HXM335	Baikeheimai	TT	2	2	red
HXM336	Baizhamang	TT	1	1	red
HXM337	Baiquanmang	TT	3	1	red
HXM338	Baiyouzitou	TT	3	2	red
HXM339	Baishenmai	TT	1	1	red
HXM340	Youmanggedacao	CC	3	1	red
HXM341	Huitoucao	TT	2	1	red
HXM342	Guangtoumai	CC	1	2	red
HXM343	Hongmangbai(Xihua)	CC	5	4	red
HXM344	Hongmanghong(Xuchang)	TT	5	4	red
HXM345	Hongmangmai(Wuyang)	TT	1	1	red
HXM346	Hongmangmai(Changge)	TT	2	2	red
HXM347	Hongmangshen	TT	1	2	red
HXM348	Hongmangben	TT	3	1	red
HXM349	Hongmangcao(Xihua)	TT	3	2	red
HXM350	Hongmangcao(Yanling)	CC	1	2	red

HXM351	Hongheshangtou(Lushan)	TT	1	2	red
HXM352	Hongyouzitou(Jiaxian)	CC	3	1	red
HXZ353	Hongyouzitou(Lushan)	CC	2	1	red
HXZ354	Hongsuimai	TT	2	1	red
HXZ355	Yuanzicao	TT	3	1	red
HXZ356	Tutoumai(Yexian)	TT	2	1	red
HXZ357	Yulincao(Xiangcheng)	TT	3	1	red
HXZ358	Gedacao(Xiangcheng2)	TT	2	2	red
HXZ359	Hulutou	TT	1	2	red
HXZ360	Nanyanghong	TT	3	1	red
HXZ361	Quanmang	TT	2	1	red
HXZ362	Quanmangmai(Xihua)	TT	1	2	red
HXZ363	Quanmangcao	TT	1	2	red
HXZ364	Huangguaxian(Xihua)	TT	3	1	red
HXZ365	Huangguaxian(Jiaxian)	TT	3	1	red
HXZ366	Youzitou(Yanling)	TT	2	1	red
HXZ367	Youzitou(Wuyang)	TT	1	2	red
HXZ368	Zigancao(Xuchang)	TT	3	1	red
HXM369	Ziboyan (1)	TT	3	1	red
HXM370	Hunanbai	CC	4	5	white
HXM371	Hunanhong	TT	1	2	red
HXM372	Pushanba(Jiaxian)	TT	3	1	red
HXM373	Pushanba(Yexian)	TT	1	1	red
HXM374	Pingyuan 50	TT	1	1	red
HXM375	Youzimai(Qingfeng)	CC	4	5	white
HXM376	Xuzhou 438	CC	2	4	white
HXM377	Kaifeng 124	TT	2	2	red
HXM378	Biyumai	CC	5	3	white
HXM379	Danmai 1	CC	3	2	red
HXM380	Bima 1	CC	5	5	white
HXM381	Bima 4	CC	5	4	white
HXM382	Bima 6	CC	3	3	white

HXM383	Xinong 6028	CC	5	2	white
HXM384	Nanda 2419	CC	5	4	red
HXM385	Zaoyangmai	TT	2	2	red
HXM386	Xuzhou 8	CC	5	4	white
HXM387	Zhengzhou 15	CC	5	5	white
HXM388	Zhengzhou 24	CC	5	5	white
HXM389	Jinan 4	CC	5	4	white
HXM390	Afu	TT	3	3	red
HXM391	Abo	CC	3	3	red
HXM392	Neixiang 36	CC	4	4	white
HXM393	Neixiang 5	CC	4	4	white
HXM394	Bonong 7422	CC	2	4	white
HXM395	Tiegancao	TT	3	2	red
HXM396	Xiannong 39	TT	1	1	red
HXM397	Mala	TT	3	3	red
HXM398	Gaojiasuo	CC	2	2	red
HXM399	Shanqianmai	TT	3	3	red
HXM400	Luofulin 10	TT	2	3	red
HXM401	Aimengniu	CC	5	5	white
HXM402	Fulanni	CC	2	3	white
HXM403	Zhaomai 2	CC	2	3	white
HXM404	Zhengmai 6	CC	5	5	white
HXM405	Zhengmai 5	CC	2	3	white
HXM406	Boai 7023	TT	3	1	red
HXM407	Zhengzhou 683	TT	2	4	red
HXM408	Zhengyin 1	CC	2	3	red
HXM409	Zhengyin 4	TT	4	3	red
HXM410	Xuzhou 14	CC	3	3	white
HXM411	Puyang 5	CC	5	5	white
HXM412	Anxuan 5	CC	5	5	white
HXM413	Yanda 25	CC	4	5	white
HXM414	Xiaoyan 4	CC	3	4	white

HXM415	Zhengzhou 17	CC	5	5	white
HXM416	Zhengzhou 761	CC	5	5	white
HXM417	Zhengliufu	CC	5	5	white
HXM418	Aifeng 3	CC	5	5	white
HXM419	Zhengzhou 722	TT	3	2	red
HXM420	Fengchan 1	CC	2	3	white
HXM421	Fengchan 3	CC	3	3	white
HXM422	Xiaoyan 6	CC	1	2	red
HXM423	Yanshi 4	CC	5	3	red
HXM424	Xian 8	CC	4	4	white
HXM425	Bainong 3217	CC	3	2	red
HXM426	Yumai 1	TT	2	3	red
HXM427	Yumai 2	CC	4	3	red
HXM428	Yumai 4	CC	5	4	white
HXM429	Yumai 5	CC	4	5	white
HXM430	Yumai 6	CC	5	3	white
HXM431	Yumai 7	CC	5	5	white
HXM432	Yumai 8	CC	5	5	white
HXM433	Yumai 10	CC	5	5	white
HXM434	Yumai 12	CC	5	5	white
HXM435	Yumai 13	TT	3	3	white
HXM436	Xuzhou 21	CC	5	5	white
HXM437	Shannong 7859	CC	4	3	white
HXM438	Een 1	TT	2	2	red
HXM439	Zhengtaiyu 1	CC	5	3	red
HXM440	Yumai 14	CC	5	5	white
HXM441	Yumai 15	CC	5	5	white
HXM442	Yumai 16	CC	5	5	white
HXM443	Yumai 17	CC	5	5	white
HXM444	Yumai 18	CC	5	5	white
HXM445	Yumai 19	CC	5	5	white
HXM446	Yumai 21	CC	5	5	white

HXM447	Yumai 24	CC	4	4	white
HXM448	Yumai 25	CC	5	5	white
HXZ449	Yumai 26	CC	5	5	white
HXZ450	Yumai 28	CC	5	5	white
HXZ451	Yumai 29	CC	5	5	white
HXZ452	Yumai 30	CC	5	5	white
HXZ453	Yumai 32	CC	5	5	white
HXZ454	Yumai 33	TT	3	3	white
HXZ455	Yumai 34	CC	5	5	white
HXZ456	Yumai 35	CC	5	5	white
HXZ457	Yumai 36	CC	5	5	white
HXZ458	Yumai 40	CC	5	4	white
HXZ459	Yumai 41	CC	5	5	white
HXZ460	Yumai 43	CC	5	4	white
HXZ461	Zhongyu 3	CC	5	4	white
HXZ462	Yumai 45	CC	5	5	white
HXZ463	Yumai 47	CC	5	5	white
HXZ464	Yumai 48	CC	2	5	white
HXM465	Yumai 49	CC	5	5	white
HXM466	Yumai 50	CC	5	5	white
HXM467	Yumai 51	CC	5	5	white
HXM468	Yumai 52	CC	5	4	white
HXM469	Yumai 54	CC	5	5	white
HXM470	Yumai 55	CC	3	5	white
HXM471	Yumai 56	CC	5	5	white
HXM472	Yumai 57	CC	5	4	white
HXM473	Yumai 58	CC	5	5	white
HXM474	Yumai 59	CC	5	5	white
HXM475	Yumai 60	CC	5	5	white
HXM476	Yumai 61	CC	5	5	white
HXM477	Yumai 62	CC	5	5	white
HXM478	Yumai 63	CC	5	5	white

HXM479	Yumai 64	CC	5	5	white
HXM480	Yumai 67	CC	5	5	white
HXM481	Yumai 68	CC	5	5	white
HXM482	Yumai 69	CC	5	5	white
HXM483	Yumai 70	CC	5	4	white
HXM484	Zhongyu 5	CC	5	4	white
HXM485	Zhongyu 6	CC	5	5	white
HXM486	Zhoumai 11	CC	4	5	white
HXM487	Luomai 1	CC	5	5	white
HXM488	Luohan 2	CC	5	5	white
HXM489	Luohan 3	CC	5	5	white
HXM490	Zhengmai 9023	CC	5	5	white
HXM491	Aikang 58	CC	3	5	white
HXM492	Zhengmai 366	CC	5	5	white
HXM493	Zhengmai 004	CC	5	5	white
HXM494	Yumai 70-36	CC	5	5	white
HXM495	Zhoumai 13	CC	5	5	white
HXM496	Zhoumai 16	CC	5	5	white
HXM497	Zhoumai 17	CC	5	5	white
HXM498	Zhoumai 18	CC	5	5	white
HXM499	Xuke 718	CC	5	4	white
HXM500	Xuke 316	CC	5	5	white
HXM501	Yumai 18-99	CC	5	2	white
HXM502	Fanmai 5	CC	3	5	white
HXM503	Xinmai 12	CC	5	5	white
HXM504	Xinmai 13	CC	3	4	white
HXM505	Xinmai 16	CC	3	4	white
HXM506	Xinmai 18	CC	5	4	white
HXM507	Yanzhan 4110	CC	5	5	white
HXM508	Jimai 1	CC	5	5	white
HXM509	Xinmai 11	CC	5	5	white
HXM510	Wan 369	CC	5	5	white

HXM511	Taikong 6	CC	5	5	white
HXM512	Pumai 9	CC	3	4	white
HXM513	Zhongyuan 98-68	CC	5	5	white
HXM514	Xinmai 208	CC	5	5	white
HXM515	Yumai 49-198	CC	5	4	white
HXM516	Zhengmai 9405	CC	5	5	white
HXM517	Zhengmai 9694	CC	5	3	white
HXM518	Zhengmai 7698	CC	5	5	white
HXM519	Zhengmai 0856	CC	5	5	white
HXM520	Zhengmai 0943	CC	5	5	white
HXM521	Zhengmai 379	CC	5	5	white
HXM522	Zhengmai 3596	CC	5	5	white
HXM523	Luomai 21	CC	5	5	white
HXM524	Luomai 24	CC	5	3	white
HXM525	Luohan 6	CC	5	5	white
HXM526	Luohan 10	CC	5	4	white
HXM527	Luohan 12	CC	4	4	white
HXM528	Xinmai 19	CC	4	5	white
HXM529	Xinmai 23	CC	5	5	white
HXM530	Xinmai 26	CC	5	5	white
HXM531	Huapei 3	CC	5	5	white
HXM532	Huapei 8	CC	5	4	white
HXM533	Zhoumai 19	CC	5	5	white
HXM534	Zhoumai 20	CC	5	5	white
HXM535	Zhoumai 22	CC	5	3	white
HXM536	Zhoumai 23	CC	5	5	white
HXM537	Zhoumai 24	CC	4	5	white
HXM538	Zhoumai 26	CC	5	5	white
HXM539	Zhoumai 27	CC	5	5	white
HXM540	Zhoumai 32	CC	5	5	white
HXM541	Fengdecunmai 1	CC	5	5	white
HXM542	Zhongyu 9	CC	5	5	white

HXM543	04 zhong 36	CC	5	5	white
HXM544	Zhongyu 12	CC	5	5	white
HXZ545	Kaimai 18	CC	5	5	white
HXZ546	Jimai 19	CC	3	5	white
HXZ547	Jimai 20	CC	2	3	white
HXZ548	Jimai 22	CC	5	5	white
HXZ549	Pingan 6	CC	5	4	white
HXZ550	Pingan 8	CC	5	5	white
HXZ551	Kaimai 21	CC	5	5	white
HXZ552	Bainong 207	CC	5	5	white
HXZ553	Zhongmai 1	CC	5	4	white
HXZ554	Hengguan 35	CC	5	5	white
HXZ555	Kaimai 20	CC	5	5	white
HXZ556	Yangao 21	CC	5	5	white
HXZ557	Xinong 979	CC	5	5	white
HXZ558	Luomai 9	CC	5	5	white
HXZ559	Zhongluotiegan	CC	5	5	white
HXZ560	Xuke 1	CC	5	5	white
HXM561	Zhengyumai 9987	CC	5	5	white
HXM562	Luomai 22	CC	5	4	white
HXM563	Luomai 23	CC	5	5	white
HXM564	Xinmai 21	CC	5	5	white
HXM565	Bainong 419	CC	5	5	white
HXM566	R 93127	CC	5	5	white
HXM567	Zhengmai 1348	CC	5	5	white
HXM568	Yulong 1325	CC	5	5	white
HXM569	07H508	CC	5	5	white
HXM570	Zhengmai 1354	CC	5	4	white
HXM571	Zhengmai 1345	CC	5	5	white
HXM572	Zhengmai 1342	CC	5	5	white
HXM573	4B269	CC	5	5	white
HXM574	Zhou 8425B	CC	5	5	white

HXM575	Zhengzi07H508-1	CC	5	5	white
HXM576	Zhengzi07H508-2	CC	5	5	white
HXM577	Zhengzi07H508-3	CC	5	5	white
HXM585	Zhengmai 883	CC	5	5	white
HXM586	Zhoumai 28	CC	5	5	white
HXM587	Zhoumai 31	CC	5	5	white
HXM588	Xinmai 26-5	CC	5	5	white
HXM589	Xinmai 29	CC	5	5	white
HXM590	Xinmai 30	CC	2	5	white
HXM591	Chunmai 5	CC	5	5	white
HXM592	Chunmai 8	CC	5	5	white
HXM593	Zhongyu 1023	CC	4	5	white
HXM594	Zhongyu 9307	CC	5	5	white
HXM595	Zhongyu 1220	CC	5	5	white
HXM596	Zhongyu 1311	CC	5	5	white
HXM597	Zhongyu 1326	CC	5	5	white
HXM598	Gaoyou 8	CC	5	5	white
HXM599	Gaoyou 9828	CC	5	5	white
HXM600	Gaoyou 5766	CC	5	4	white
HXM601	Gaoyou 2018	CC	3	2	white
HXM602	Gaoyou 5218	CC	5	3	white
HXM603	Shiluan 02-1	CC	5	4	white
HXM604	Shiluan 08-4	CC	5	5	white
HXM605	Jichuang 2	CC	4	5	white
HXM606	Zhengpinmai 7	CC	5	5	white
HXM607	Luomai 28	CC	5	5	white
HXM608	Luomai 29	CC	4	5	white
HXM609	Luomai 31	CC	5	5	white
HXM610	Guomai 301	CC	5	5	white
HXM611	Huaimai 30	CC	4	5	white
HXM612	Luomai 26	CC	5	5	white
HXM613	Xuke 168	CC	5	5	white

HXM614	Bonong 6	CC	5	5	white
HXM615	Luomai 8	CC	5	5	white
HXM616	Xinmai 30	CC	3	3	white
HXM617	Guomai 0319	CC	5	5	white
HXM618	Zhengyumai 043	CC	5	5	white
HXM619	Kaimai 22	CC	5	5	white
HXM620	Wanmai 98	CC	5	5	white
HXM621	Zhongmai 875	CC	5	5	white
HXM622	Zhengmai 119	CC	5	5	white
HXM623	Zhengmai 1867	CC	5	4	white
HXM624	Zhengzi08H250-1	CC	5	4	white
HXM625	Zhengzi08H250-2	CC	5	4	white
HXM626	Zhengzi08H250-3	CC	5	5	white
HXM628	Zhengzi08H250-5	CC	5	5	white
HXM629	Zhongmai 895	CC	5	5	white
HXM632	Sulianzaoshu 1	TT	1	2	red
HXM634	Jimai 30	CC	5	5	white
HXM635	Fangtoumai	CC	1	3	red
HXM636	Yumai 65	CC	5	5	white
HXM637	Baiquan 3039	CC	4	2	white
HXM638	Baiquan 41	CC	5	3	white
HXM639	Shiai 1	CC	5	4	red
HXM640	Zhengzhou 871	CC	5	4	white

Table S4. 629 natural populations variance analysis of for PHS resistance

Source of variance	DF	SS	MS
Genotype (G)	628	108.219	0.172**
Years (Y)	1	41.963	41.963**
Genotype × Years (G×Y)	627	66.257	0.106**
Error	629	11.507	0.018
Total	1257	119.726	

**: The 0.001 probability level of significance.

Table S5. Haplotype analysis for PHS resistance at QSS.TAF9-3D locus

Haplotype	SS_2021	SS_2022
CC	3.796 ± 0.081	3.898 ± 0.071
TT	2.088 ± 0.078**	2.021 ± 0.068**
Phenotypic variation (%)	36.39	45.85

**: The 0.001 probability level of significance.

SS_2021 and SS_2022: The PHS resistance indices in 2020-2021 and 2021-2022, respectively.

Table S6. 11 PHS resistance white-grained varieties with haplotype QSS.TAF9-3D-TT

Name of variety	Haplotype	SS_2021	SS_2022
Qisiniu	TT	3	3
Yaerzui	TT	3	3
Heshangtou(Anyang)	TT	3	3
Shuanghoumai	TT	3	3
Zhuganqing(Tangxian)	TT	3	3
Baihulutou	TT	3	3
Xiaobaimai(Mianchi)	TT	3	3
Baipimai	TT	1	3
Baijuanmang	TT	3	1
Yumai 13	TT	3	3
Yumai 33	TT	3	3

SS_2021 and SS_2022: The PHS resistance indices in 2020-2021 and 2021-2022, respectively.

1 to 5: Highly resistance, resistance, middle resistance, susceptibility, and highly susceptibility, respectively.
